# Characterizing a new rat model of chronic pain after spine surgery

**DOI:** 10.1038/s41413-025-00408-1

**Published:** 2025-03-12

**Authors:** Qichao Wu, Neil C. Ford, Shaoqiu He, Chi Zhang, Xiang Cui, Jing Liu, Xueming Chen, Xu Cao, Yun Guan, Lei Zang

**Affiliations:** 1 Department of Anesthesiology and Critical Care Medicine, Johns Hopkins University, School of Medicine, Baltimore, MD USA; 2 Department of Orthopedics, Beijing Chaoyang Hospital, Capital Medical University, Beijing, China; 3 Department of Orthopedics, Beijing Luhe Hospital, Capital Medical University, Beijing, China; 4 Department of Orthopedics, Johns Hopkins University, School of Medicine, Baltimore, MD USA; 5 Department of Neurological Surgery, Johns Hopkins University, School of Medicine, Baltimore, MD USA

**Keywords:** Neurophysiology, Bone

## Abstract

Chronic pain after spine surgery (CPSS) is a complex disorder characterized by multifactorial pathogenesis that occurs in 8%–40% of patients undergoing lumbar spine surgery. We aimed to develop a rat model that mimics clinical CPSS conditions by taking two sequential surgical procedures. Step 1: A plastic rod was inserted into the left L5 intervertebral foramen to produce a steady compression on the dorsal root ganglion (DRG) and the spinal nerve, a common cause of low back pain (LBP). Step 2: The rod was removed after 7 days when rats exhibited mechanical and heat hypersensitivity in the ipsilateral hindpaw, followed by a full L5 laminectomy to mimic spine decompression surgery in LBP patients. The retention of the rod induced a prolonged LBP-like behavior but was quickly resolved after rod removal without laminectomy. However, rats that received laminectomy after rod removal developed heightened mechanical and heat sensitivity in the hindpaw, impaired gait, and reduced spontaneous exploration activity, indicating CPSS. Patch clamp recording revealed a significant augmentation in the intrinsic excitability of small-diameter DRG neurons in CPSS rats. Administration of Dermorphin [D-Arg2, Lys4] (1–4) amide (DALDA, 5 mg /kg, i.p.), a peripherally acting mu-opioid receptor (MOR)-preferred agonist, attenuated pain hypersensitivity, capsaicin-induced [Ca^2+^]i rising and the increased intrinsic excitability of DRG neurons from CPSS rats. Our findings suggest that this new model, which mirrors the nature of CPSS developed in patients, may be useful for future studies of the underlying mechanisms.

## Introduction

Low back pain (LBP) constitutes a prevalent and burdensome health condition.^1^ Surgical intervention targeting the lumbar spine represents a common therapeutic approach for LBP, which becomes resistant to conservative management strategies. Unfortunately, 8%–40% of patients after lumbar spine surgery still experience intractable back pain and/or radiating leg pain,^2,3^ which was previously termed failed back surgery syndrome. However, emerging evidence indicates that these pain symptoms persist even among patients who have undergone successful surgery or exhibit no postoperative radiographic evidence of neural tissue compression.^4^ Consequently, this chronic pain condition was reclassified as chronic pain after spine surgery (CPSS), in line with the International Classification of Diseases-11 criteria.^4^

The pathogenesis of CPSS is multifactorial and involves both structural and non-structural factors.^3^ Structural factors include ligaments, intervertebral discs, bones, fibrosis, and the postoperative epidural scar, which may cause mechanical pressure, compression, or irritation of the sensory ganglion or nerve roots.^2^ The non-structural factors encompass various postoperative changes in the local microenvironment, including inflammation, priming and sensitization of primary sensory neurons in the dorsal root ganglion (DRG) by preexisting LBP conditions, activation of glial cells, and psychological factors.^3^ Presently, the precise mechanisms for the initiation and progression of CPSS remain insufficiently elucidated.

A lack of clinically pertinent models represents a limiting factor in the mechanistic studies of CPSS.^3^ In essence, two critical conditions need to be met for a CPSS model to replicate clinical scenarios. The LBP condition should be first established, often by compression of the ganglion and/or nerve roots. Once LBP is developed, a laminectomy needs to be performed to mimic decompression spine surgery in patients. However, most CPSS models only involve full laminectomy or hemilaminectomy in the thoracic or lumbar spine vertebrae in naive animals. The resulting post-laminectomy pain is short-lasting, without the initial establishment of the LBP condition.^5,6^ Although one study explored CPSS mechanisms by combining laminectomy and bilateral cutting of the L4-L5 dorsal roots,^7^ acute dorsal root rupture is rare in clinical practice.

To our knowledge, there are presently no clinically pertinent CPSS models, especially for investigating its non-structural mechanisms. Therefore, we set to develop a CPSS model to better simulate the clinical scenario by conducting decompression laminectomy surgery in rats with preexisting LBP. We first examined whether these rats showed prolonged pain hypersensitivity, reduced spontaneous exploration, and abnormal gait. We then conducted patch clamp recording to examine changes in the intrinsic excitability of small-diameter DRG neurons in CPSS rats. Finally, we tested whether Dermorphin [D-Arg2, Lys4] (1–4) amide (5 mg /kg, i.p.), a peripherally acting MOR-preferred agonist which inhibited neuropathic pain,^8,9^ can also attenuate pain and capsaicin-induced increases in [Ca^2+^]i in DRG neurons from CPSS rats. We postulate that this new model will mirror some of the natures of CPSS in humans and be suitable for studying its underlying mechanism in the future.

## Results

### Changes in evoked pain sensitivity and locomotor function after chronic DRG compression

**Fig. 1:**
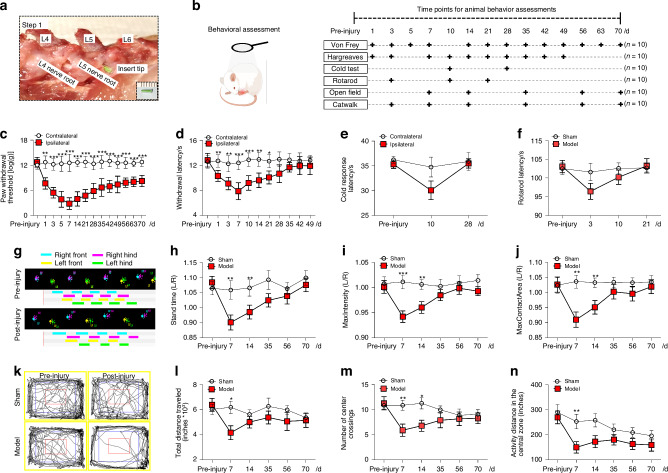
Changes in evoked pain responses and locomotor function after chronic dorsal root ganglion (DRG) compression. **a** An image illustrating the compression of the L5 dorsal root ganglia (DRG) and spinal nerve by inserting and retaining the plastic rod (diameter: 0.8–1 mm, length: 6 mm). **b** Diagram of the experimental protocol. **c** Changes in paw withdrawal threshold (PWT) to mechanical stimuli on the ipsilateral hind paw after DRG compression, compared to the contralateral hind paw (*n* = 10). **d** Changes in paw withdrawal latency (PWL) to heat stimuli following surgery (*n* = 10). **e** Changes in paw withdrawal latency (PWL) to cold stimuli (*n* = 10). **f** In the rotarod test, there were no significant differences in fall latency between surgery (*n* = 10) and sham-operated groups (*n* = 10). **g** Representative images of CatWalk assay. **h**–**j** Gait analysis revealed that rats exhibited significantly shorter stand time, lower max-intensity, and smaller max-contact area than sham-operated rats on postoperative day 7 (*n* = 10). **k** Representative images of the open field test. In the open-field test, significant differences were observed in the total distance traveled (**l**), number of center crossings (**m**), and activity distance in the central zone (**n**) on postoperative day 7 between the model and sham-operated rats (*n* = 10/group). *P < 0.05, **P < 0.01, ***P < 0.001, ipsilateral hind paw vs contralateral hind paw or sham group vs model group. Two-way mixed model ANOVA followed by Sidak’s multiple comparisons test. Data are expressed as mean ± SEM. Note: The horizontal time axis is not equally spaced

Mechanical, heat, and cold sensitivities were assessed in rats before and after inserting a rod into the L5 intervertebral foramen and retaining it till the experiment ended (Fig. 1a, b), which has been used for developing LBP model.^10^ PWT in response to mechanical stimuli showed a prolonged decrease on the ipsilateral hindpaw, compared to the contralateral side. The most notable reduction occurred in postoperative weeks 1 and 2 (Fig. 1c). PWL to radiant heat stimulation was also significantly decreased following surgery, reaching its maximum decrease on postoperative day 7, and gradually returned to a level comparable to the contralateral hind paw over 28 days (Fig. 1d). There was a trend towards the decreased ipsilateral PWL to cold stimuli at postoperative day 10, but this difference did not attain statistical significance (Fig. 1e).

We also employed three tests to evaluate changes in locomotor function. In the rotarod assay to measure motor coordination and balance,^11^ a trend of decreased fall latency was noted in the surgery group, but the difference was not significant compared to the sham-operated group (Fig. 1f). In the CatWalk assay to evaluate the gait patterns of animals in an unforced, voluntary movement setting (Fig. 1g), rats exhibited significantly shorter stand time (Fig. 1h), lower max-intensity (Fig. 1i), and smaller max-contact area (Fig. 1j) at postoperative days 7–14, compared to sham-operated rats. In the open-field test to assess spontaneous locomotor activity and exploratory behavior in a novel environment (Fig. 1k), rats showed significant decreases in total distance traveled (Fig. 1l), number of center crossings (Fig. 1m), and activity distance in the central zone (Fig. 1n) at postoperative day 7, compared to sham-operated group.

### Effects of rod removal with and without laminectomy on pain hypersensitivity and impaired locomotor function after DRG compression

**Fig. 2:**
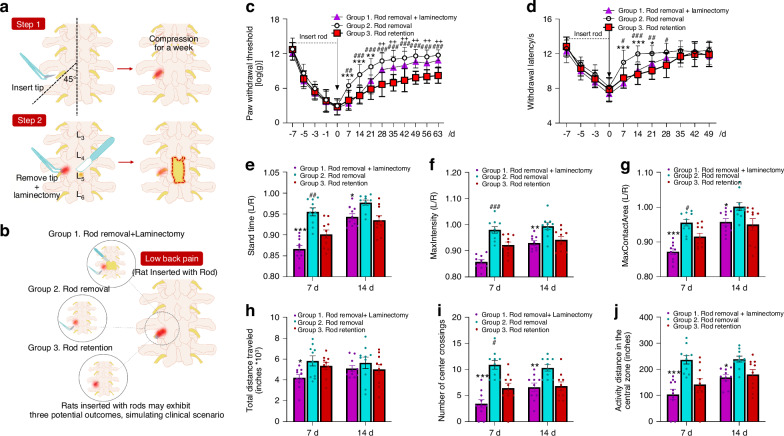
Changes in pain sensitivity and locomotor function after dorsal root ganglion (DRG) compression, followed by decompression with and without a laminectomy. **a** Schematic diagram of establishing the CPSS model. The intervertebral foramen on the left side of the L5 vertebra was exposed, and a small plastic rod (diameter: 1 mm; length: 6 mm) was inserted to induce a chronic steady compression of the DRG and spinal nerve. After 7 days, the rod is removed, followed by partial L5 laminectomy to mimic surgical intervention for decompression. **b** The schematic diagram illustrates the three interventions: rod removal with laminectomy, rod removal, and rod retention, conducted on day 7 after rod insertion. **c** Changes in paw withdrawal threshold (PWT) to mechanical stimuli on the ipsilateral hind paw in each group (*n* = 10/group). **d** Changes in paw withdrawal latency (PWL) to heat stimuli following surgery (*n* = 10/group). **e**–**g** The standing time, maximum intensity, and maximum contact area of Catwalk analysis in each group at 7 and 14 days after intervention (*n* = 10/group). **h**–**j** Total distance traveled, number of center crossings, and activity distance in open-field test in each group 7 and 14 days after intervention (*n* = 10/group). Two-way mixed model ANOVA followed by Sidak’s multiple comparisons test. *P < 0.05, **P < 0.01, ***P < 0.001, Group 1 (rod removal with laminectomy) vs Group 2 (rod removal). ^#^*P* < 0.05, ^##^*P* < 0.01, ^###^*P* < 0.001, Group 2 (rod removal) vs Group3 (rod retention). ^+^*P* < 0.05, ^++^*P* < 0.01, ^+++^*P* < 0.001, Group 1 (rod removal with laminectomy) vs Group 3 (rod retention). Data are expressed as mean ± SEM. Note: The horizontal time axis in (**c**, **d**) is not equally spaced

Based on the above findings, we then examined the effects of rod removal, with and without laminectomy, on established pain hypersensitivity in rats that had received rod insertion. *Group 1* (Rod removal + Laminectomy, CPSS): To replicate spinal surgical intervention to alleviate LBP in patients, the inserted rod was removed on day 7 after Step 1 implantation surgery, followed by a full laminectomy at the L5 spinal level to mimic the decompression surgery (Fig. 2a). Rod removal with laminectomy emulates the comprehensive clinical decompression of the dura mater and nerve roots. *Group 2* (Rod removal only): The second group of LBP rats received only rod removal, including the midline skin incision and paraspinal muscle separation at day 7 after Step 1 implantation surgery, but without laminectomy (Fig. 2b). This procedure may be regarded as a form of micro-decompression of the nerve tissue, akin to minimally invasive techniques employed in clinical practice. It focuses on localized decompression while minimizing the extensive tissue disruption typically associated with traditional laminectomy. *Group 3* (Rod retention): The third group was the LBP rats shown in Fig. 1; they received neither rod removal nor laminectomy after Step 1 surgery. Since they were used to mimic the conservative treatment without decompression, Group 3 rats did not receive skin incision and paraspinal muscle separation.

All three groups of rats developed comparable mechanical and heat hypersensitivity in the ipsilateral hindpaw on day 7 after rod implantation, before Step 2 interventional surgery (day 0, Fig. 2c, d). In Group 1, the decreased PWT and PWL gradually returned toward baseline level (i.e., before rod implantation, day −7) after the intervention. Compared to Group 1, the recovery from mechanical and heat hypersensitivities in Group 2 rats, which received only rod removal, was sooner and greater (Fig. 2c, d). The PWT and PWL during the initial 1–2 weeks in Group 2 after intervention were significantly higher than that in the other two groups (Fig. 2c, d). In contrast, the recovery from mechanical hypersensitivity was much less and slower in Group 3 rats, which received no intervention (i.e., rod retention), indicating prolonged pain hypersensitivity due to chronic compression. Notably, rats undergoing both rod removal and laminectomy exhibited lower PWT and PWL at days 7 and 14 after the intervention, compared to those undergoing rod removal alone, indicating the presence of CPSS. Over time, their pain sensitivity gradually ameliorated, reaching comparable levels by day 28 after intervention.

In the Catwalk analysis, Group 1 rats that underwent both rod removal and laminectomy also displayed less improvement in standing time (Fig. 2e), a lower maximum intensity (Fig. 2f), and maximum contact area (Fig. 2g) at days 7 and 14 after the intervention, compared to Group 2 rats undergoing rod removal alone. Group 2 rats also showed more improvement on day 7 post-intervention, compared to that in Group 3 rats. The findings from open field tests agreed with the above findings. Group 1 rats displayed lower total distance traveled (Fig. 2h), number of center crossings (Fig. 2i), and activity distance (Fig. 2j), compared to Group 2.

Collectively, these findings indicate that rats with existing LBP and subjected to both rod removal and laminectomy still exhibit significant mechanical and heat hypersensitivity and functional impairment, which may mimic certain features of CPSS in patients after decompression procedures. Further analysis found no gender difference in pain behavior changes in Group 1 rats (i.e., CPSS model). The ipsilateral PWT and PWL (Fig. S1A, B), the standing time, maximum intensity, and maximum contact area in Catwalk analysis (Fig. S1C–F) were comparable between male and female rats at day 7 and 14 after Step 2 surgery. To rule out the possibility of nerve tissue damage due to laminectomy, which may also induce pain, we conducted laminectomy procedures in naive rats. Laminectomy alone did not significantly change the PWT and PWL at days 7 and 14 after surgery, compared to naïve rats (Fig. S2).

### Enhanced intrinsic excitability of small-diameter DRG neurons in CPSS rats

**Fig. 3:**
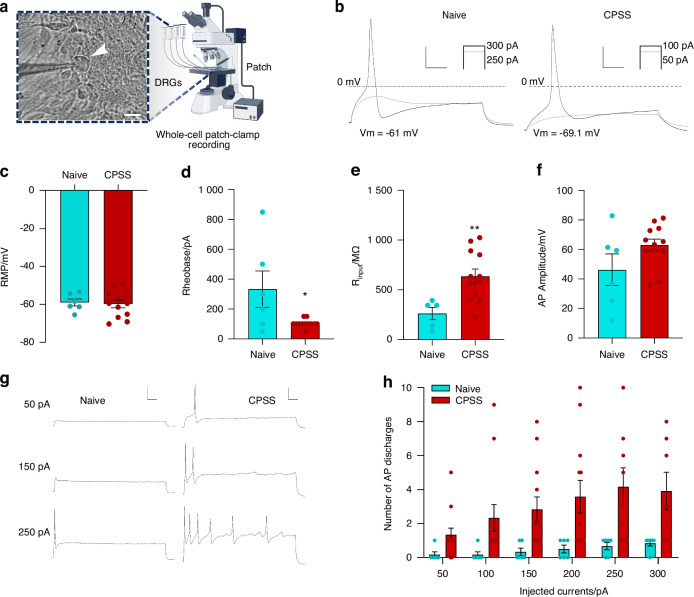
Increased intrinsic excitability of small-diameter DRG neurons after CPSS. **a** Bright-field image depicting a recorded small-diameter DRG neuron. Scale bar: 10 µm. **b** Representative rheobase traces from small-diameter DRG neurons under naive and CPSS conditions. The ipsilateral L5 DRG was harvested in rats (10 weeks, 250–300 g) with rod insertion on day 7 after Step 2 surgery. Scale bar: 40 mV, 20 ms. **c** CPSS did not affect the resting membrane potential (RMP) of small-diameter DRG neurons [(12) = 1.21, *P* = 0.24; unpaired *t*-test]. **d** CPSS significantly reduced the rheobase levels in nociceptive DRG neurons [t(12) = 2.22; *P = 0.04], unpaired *t*-test. **e** Input resistance was elevated in DRG neurons from CPSS [t(11) = 3.9; **P = 0.002], unpaired *t*-test. **f** The mean action potential (AP) amplitude evoked by 1 000 pA current injection was increased in CPSS DRG neurons but did not reach significance [t(12) = 1.78; *P* = 0.1], unpaired *t*-test, compared to the naïve group. **g** Representative DRG neuron firing traces to the current injection at 50, 150, and 250 pA in cultured DRG neurons from naive and CPSS rats. Scale bars: 20 mV, 0.1 s. **h** There was a trend that small-diameter DRG neurons in the CPSS group showed an increased mean firing frequency evoked by increasing intensities of current injections, compared to the naive group [F(5,60) = 0.58; *P* = 0.7]. Two-way mixed model ANOVA followed by Sidak’s multiple comparisons test. Data are expressed as mean ± SEM

Since noxious inputs are transmitted mostly by small-diameter DRG neurons, we then conducted patch clamp recording to assess the intrinsic excitability of small-diameter DRG neurons in rats with CPSS (Fig. 3a, b). The ipsilateral L5 DRG was harvested in rats (10 weeks, 250–300 g) with rod insertion on day 7 after Step 2 surgery and in naïve rats. The resting membrane potential of small-diameter DRG neurons was comparable between the two groups (Fig. 3c). However, the rheobase level to evoke action potential (AP) was significantly lowered in CPSS rats, compared to naïve rats (Fig. 3d, g). The input resistance was significantly increased in the CPSS group (Fig. 3e). There was a trend that the AP amplitude (Fig. 3f) and the mean AP firing rate (Fig. 3h) were also increased in the CPSS group, compared to naive rats. These findings suggest a heightened intrinsic excitability of DRG neurons in CPSS rats.

### DALDA attenuated mechanical and thermal hypersensitivity and reduced intrinsic excitability in DRG neurons in CPSS rats

**Fig. 4:**
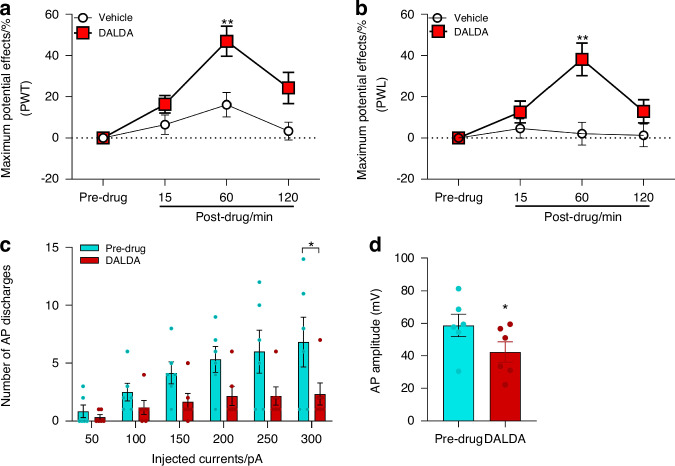
DALDA attenuates mechanical and heat hypersensitivity while reducing the intrinsic excitability in DRG neurons from CPSS rats. **a** At day 7 after Step 2 surgery, intraperitoneal (i.p.) administration of DALDA (5 mg/kg) attenuated the mechanical hypersensitivity in the ipsilateral hind paws of CPSS rats 60 min post-administration, as indicated by increased paw withdrawal threshold (PWT) from baseline (*n* = 10/group). **b** DALDA (5 mg/kg) also attenuated heat hypersensitivity in the ipsilateral hind paws of CPSS rats, as indicated by increased paw withdrawal latency (PWL) from baseline (*n* = 10/group). **c** In the patch-clamp recording, DALDA treatment (5 μmol/L, bath application, *n* = 6) reduced action potential (AP) discharge evoked by increasing current stimulation in cultured DRG neurons from CPSS rats. The ipsilateral L5 DRG was harvested in rats (10 weeks, 250–300 g) with rod insertion on day 7 after Step 2 surgery. **d** DALDA (5 μmol/L, bath application, *n* = 6) also reduced the mean AP amplitude evoked by 300 pA current stimulation in these neurons. *P < 0.05, **P < 0.01, vs pre-drug. Data are expressed as mean ± SEM. **a**–**c** Two-way mixed model ANOVA followed by Sidak’s multiple comparisons test. **d** Paired *t*-test. Maximum Potential Effects (MPW)% = [(post-drug) – (pre-drug)]/[(baseline) – (pre-drug)] × 100%

To further validate this new model, we tested if DALDA, a peripherally activating mu-opioid receptor (MOR)-preferring agonist that inhibited neuropathic pain in rats,^12^ may also alleviate pain hypersensitivity in CPSS rats. Based on previous findings,^12^ we administered vehicle or DALDA intraperitoneally (i.p.) at a dose of 5 mg/kg in rats on day 7 after Step 2 surgery. The ipsilateral PWT and PWL in CPSS rats were significantly increased at 60 min after injection of DALDA, but not vehicle, compared to pre-drug (Fig. 4a, b), suggesting an inhibition of mechanical and thermal hypersensitivity.

**Fig. 5:**
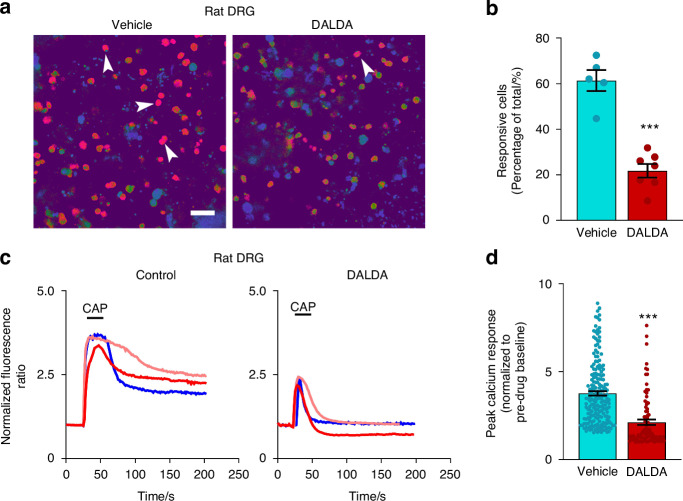
DALDA attenuates capsaicin-induced elevations in intracellular calcium levels in cultured DRG neurons derived from CPSS rats. **a** Representative images depicting calcium responses of cultured rat sensory neurons to capsaicin (CAP, 0.3 μmol/L, 20 s). Arrowheads indicate capsaicin-responsive neurons which showed a robust increase in fluorescence intensity (red color) to capsaicin treatment. L5 DRG neurons (DIV 2) removed from CPSS rats on day 7 after Step 2 surgery were loaded with the fluorescent calcium indicator Fura2-AM (2 μg/mL) and maintained at 37 °C for 45 min, followed by a 15 min de-esterification period at 37 °C in warmed external solution. The viability of neurons was confirmed at the end of the experiment by the addition of KCl (50 mmol/L), which activates all live cells. Scale bars, 100 μm. **b** Quantification of calcium-imaging assays (*n* = 5–7) revealed a significant reduction in the percentage of capsaicin-responsive neurons following DALDA treatment (5 μmol/L, bath application, 10 min, Vehicle: 61.3% ± 4.7%, DALDA: 21.8% ± 3.0%). **c** Representative traces of normalized fluorescence ratio demonstrating the inhibition of capsaicin-induced [Ca^2+^]i increase following DALDA treatment (5 μmol/L, bath application, 10 min) in dissociated DRG neurons, compared to vehicle treatment. **d** Peak calcium responses to capsaicin of individual DRG neurons with vehicle and DALDA treatment (Vehicle: 3.8 ± 0.1, DALDA: 2.1 ± 0.2; neurons from ipsilateral L5 DRG). Data are expressed as mean ± SEM. ***P < 0.001, vs vehicle. Unpaired *t*-test

Compared to pre-drug, bath application of DALDA (5 μmol/L) also significantly reduced AP firing rate and AP amplitude in cultured small-diameter DRG neurons from CPSS rats (Fig. 4c–d), suggesting an inhibition of intrinsic excitability. In calcium imaging experiments, we further assessed the impact of DALDA on the capsaicin-induced elevation of [Ca^2+^]i in DRG neurons isolated from CPSS rats. The ipsilateral L5 DRG was harvested in Sprague-Dawley rats (10 weeks, 250–300 g) with rod insertion on day 7 after Step 2 surgery. The proportion of DRG neurons responsive to capsaicin (0.3 μmol/L, 20 s) was significantly lower in DALDA-pretreated group (5 μmol/L, bath application, 10 min), as compared to that in the vehicle group (Fig. 5a, b). Moreover, pre-incubation with DALDA significantly attenuated the peak calcium response to capsaicin, as compared to vehicle pretreatment (Fig. 5c, d). These findings suggest that systemic administration of DALDA can inhibit CPSS pain and attenuate DRG neuron excitability.

### Increased expressions of MOR, GFAP, and TRPA1 in DRGs of CPSS rats

**Fig. 6:**
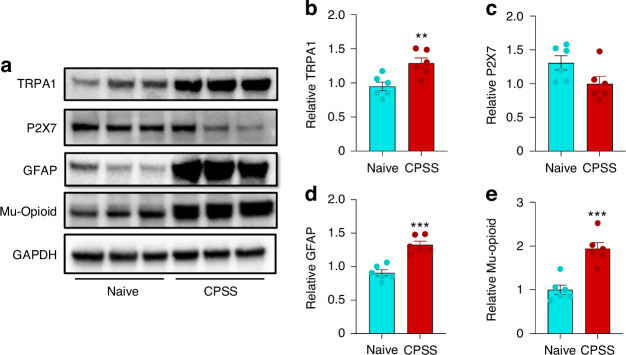
Increases in the expression of TRPA1, GFAP, and mu-opioid receptors in the ipsilateral lumbar DRGs of CPSS rats. **a** Representative Western blot analysis images. The levels of transient receptor potential ankyrin 1 (TRPA1, **b**), P2X7 receptor (**c**), glial fibrillary acidic protein (GFAP, **d**), and mu-opioid receptor (**e**) in the ipsilateral L5-6 DRGs of rats at day 7 after Step 2 surgery and in naive rats (*n* = 6/group). *P < 0.05, **P < 0.01, ***P < 0.001, vs naïve. Data are expressed as mean ± SEM. Unpaired *t*-test

Since DALDA may inhibit CPSS by activating MOR on DRG neurons, we then explored changes in the expression of some key pain-related receptors, including MOR, in CPSS rats. Due to the low protein yield from DRG tissue, we first collected the ipsilateral L5-L6 DRGs in CPSS rats (Group 1, removal of rod at day 7 after insertion followed by a full L5 laminectomy) and in naïve rats. Compared to that in naive rats, the MOR level in the DRG of CPSS rats was significantly increased (Fig. 6a). The levels of transient receptor potential ankyrin 1 (TRPA1) and glial fibrillary acidic protein (GFAP) were also significantly upregulated (Fig. 6b–e). Whereas the expression of P2X7 was not significantly different between the two groups (Fig. 6c). Given that the L5 DRG is directly impacted by rod insertion and was utilized in our functional studies, we further investigated MOR levels in the L5 DRGs of CPSS rats, which also exhibited a significant increase compared to the naïve group (Fig. S3).

## Discussion

The mechanisms underlying CPSS remain partly understood, and there is a dearth of an animal model miming clinical CPSS conditions. Here, we utilized rats initially subjected to rod insertion to induce LBP, followed by a decompression surgery with rod removal and laminectomy, as a potential clinically pertinent CPSS model. Moreover, we showed that systemic administration of DALDA exhibited efficacy in ameliorating both mechanical and thermal hypersensitivity and attenuated the increased DRG neuron excitability in CPSS rats.

### The behavioral changes in CPSS rats and clinical relevance

Although not statistically significant, there was a trend of reduced pain in rats that received rod removal and laminectomy (CPSS, Group 1) compared to rats with chronic rod retention (LBP, Group 3). Nevertheless, both CPSS and LBP mice developed long-lasting hypersensitivity to mechanical and thermal stimuli in the ipsilateral hind paw, aligning with the pain symptoms observed in these patients where individuals may experience heightened sensitivity to stimuli along one or both legs.^3,13,14^ While hypersensitivity to somatic sensory stimuli represents one aspect of CPSS, it is important to note that CPSS in humans is multifaceted, encompassing various types such as ongoing pain, background pain, spontaneous pain, and pain induced by movement. In particular, LBP and CPSS often impair a patient’s gait and mobility, as patients may compensate for pain or limited range of motion in certain parts.^15^ Intriguingly, CatWalk gait analysis showed that LBP rats also showed significantly shorter stand time, lower max-intensity, and smaller max-contact area at days 7 and 14 after rod implantation than sham-operated rats. CPSS rats at days 7 and 14 after Step 2 surgery exhibited similar, if not greater, levels of gait impairment. Additionally, in the open field test, the total distance traveled, number of center crossings, and activity distance in the central zone were significantly decreased in LBP rats at day 7 after rod insertion, as compared to sham-operated rats. Similar findings were observed in CPSS rats, suggesting an altered exploratory behavior, a form of active motor activity.

Impaired gait and decreased exploratory behavior could be due to pain, altered locomotor functions, anxiety, and stress, such as that in OA pain and neuropathic pain models.^16,17^ The LBP rats only showed a trend of impaired passive movement in the rotarod test, indicating preserved muscle strength (Fig. S4). Clinicians recognize that initial symptoms in CPSS patients often present as back or limb pain, while actual muscle strength decline typically occurs in the disease’s mid-to-late stages. Some patients exhibit early signs such as lameness, limited range of motion, and other limb movement constraints resulting from CPSS-induced pain. It remains to be determined which of the aforementioned factors is important to the impaired gait and reduced exploration in LBP and CPSS rats.

### Changes in DRG neuron excitability in CPSS rats

An increased excitability of DRG neurons is intricately linked to chronic pain and hyperalgesia.^18^ Previous studies have highlighted the impact of neuropathic pain on small-diameter DRG neurons, and our electrophysiology recording showed an increased intrinsic excitability of these neurons in CPSS rats, which could make them more responsive to peripheral stimulation, leading to pain hypersensitivity. These alterations can be triggered by inflammatory mediators,^19^ which lead to the upregulation of N- and T-type calcium channels in DRG neurons.^18^ In the LBP condition, spine surgery may intensify preexisting inflammatory responses, creating an even more pro-inflammatory environment that impacts adjacent DRGs and induces hyperalgesia and radicular pain. Clinical evidence has revealed the presence of various inflammatory mediators, including IL-6, IL-8, and prostaglandin E2 (PGE2), in wound drainage from patients who have undergone spinal surgery.^20,21^ In CPSS rats, the laminectomy may result in additional tissue damage and inflammatory responses in close proximity to the lumbar DRGs. Our findings of elevated expression of GFAP in the lumbar DRGs of CPSS rats provide indirect evidence that the pro-inflammatory environment surrounding the surgery site affects neighboring DRGs, which may partly contribute to hyperalgesia and radicular pain. Comparatively, rats in the minimally invasive decompression group (only rod removal) exhibited better behavioral outcomes, suggesting that the hyperalgesia in CPSS rats may be partly due to the extensive tissue damage caused by the laminectomy surgery under LBP condition.

Interestingly, the MOR, which mediates morphine analgesia,^22,23^ also showed an upregulation in the lumbar DRGs in CPSS rats. Importantly, intraperitoneal administration of DALDA mitigated mechanical and heat hypersensitivity in CPSS rats, with the maximum effect observed 1 h after administration. In line with this finding, patch clamp recording and calcium imaging, which are important and complementary techniques for examining neuronal excitability, showed that DALDA reduced the number and amplitude of action potential firing and suppressed capsaicin-induced increases of [Ca^2+^]i in L5 DRG neurons from CPSS rats, indicating inhibition of neuron excitability.

In the current study, our primary goal is to test whether DALDA inhibits DRG neuron responses in the CPSS model. Changes in MOR expression in the DRGs of Group 2 and Group 3 rats, which also exhibited varying degrees of pain, warrant further investigation. Nevertheless, we have previously shown that DALDA also inhibited pain behavior and DRG neuronal activity in other pain conditions (e.g., neuropathic pain).^22,24^ Therefore, it is possible that DALDA may also inhibit neuron responses in other pain groups (e.g., Group 3: Rod retention). Given that the lack of tight junctions in the endothelium of vessels supplying DRG,^25,26^ increased MOR expression in primary sensory neurons may represent a promising target for enhancing the efficacy of peripherally acting MOR agonists (e.g., DALDA) to inhibit CPSS and avoid severe side effects (e.g., sedation, addiction) associated with the activation MOR in the central nervous system.

### Advantages of the new CPSS model

In the current study, rats undergoing both rod removal and laminectomy achieved sufficient neural tissue decompression, yet their pain hyperalgesia did not ameliorate during the early post-intervention phase. This observation parallels the clinical scenario where patients, despite undergoing successful decompression procedures for LBP, continue to experience prolonged pain symptoms. This finding provides valuable insights into exploring the mechanisms of non-structural factors contributing to pain persistence. Our new CPSS model may replicate the progression of human LBP from its onset to surgical intervention, culminating in establishing a rat model exhibiting cutaneous hypersensitivity and gait impairment. As such, it may offer a potentially more faithful representation of the natural course of CPSS in humans, compared to previously described models, such as laminectomy alone, hemilaminectomy,^5,6^ or bilateral dorsal root cutting,^7^ as these prior models deviate from the clinical setting to varying extents.

CPSS stemming from identifiable structural factors typically necessitates revision surgery.^27^ Conversely, CPSS arising from non-structural factors lacks clear etiology and effective treatment options. Hence, greater emphasis should be placed on investigating non-structural factors to enhance understanding and therapeutic strategies for this condition. The preparation protocol for this animal model circumvents potential nerve tissue decompression resulting from structural factors by removing the rod and fully alleviating nerve compression via laminectomy. Yet, despite these interventions, the rats still demonstrated hyperalgesia. To rule out the possibility of nerve tissue damage due to laminectomy, we conducted laminectomy procedures in naive rats; however, they did not exhibit pain hyperalgesia. Thus, the primary advantage of this novel model may lie in its heightened clinical resemblance, particularly in mirroring the disease evolution process, and will be suitable for investigating the mechanisms associated with non-structural factors.

### limitations

Our model still faces challenges in capturing all these nuanced clinical symptoms.^28^ Although it effectively mirrors hyperalgesia and gait impairment observed in clinical settings, additional behavioral assessment techniques (e.g., spontaneous and ongoing pain) are warranted to comprehensively evaluate other aspects of CPSS. In addition, it’s widely acknowledged that lumbar spine loads are greater in humans compared to quadrupeds, presenting a common challenge for quadrupedal disease models. However, quadruped spines endure continuous anterior-posterior compression from paravertebral muscles and ligaments, thereby sustaining axial stress to some degree. The position of the inserted rod may have slightly changed during free movement. To limit this, we tightly sutured the muscle overlying the rod and observed that the rod remained in the inserted position when we conducted Step 2 surgery in Group 1 and Group 2 rats.

We observed a clear sign of compression in the ipsilateral L5 DRG after rod insertion (Fig. S5). Additionally, by staining for caspase-3, a key enzyme involved in the process of apoptosis or programmed cell death, we observed a significant increase in the percentage of caspase-3 positive neurons in the ipsilateral L5 DRGs of CPSS rats compared to the contralateral side, indicating neuronal damage (Fig. S6). We speculate that this change is more likely caused by chronic compression rather than directly injuring the DRG from the inserted rod, as we standardized and limited the insertion depth of the small blunt rod (tip diameter: 1 mm; length: 6 mm), keeping at least 2–3 mm outside the intervertebral foramen rather than fully inserting it. The etiology of clinical lower back pain is highly complex, and neural tissues are often subjected not only to mechanical compression but also to chemical irritation from intervertebral discs.^29^ This presents a key challenge for current animal models of lower back pain caused by mechanical nerve compression.

A gender disparity has been noted in CPSS diagnosis, with more women diagnosed compared to men.^30^ Yet, female and male CPSS rats exhibit similar mechanical and heat hypersensitivity. This discrepancy may be partly due to different outcome measures and species differences that may influence the progression of CPSS.^31,32^ For example, clinicians typically diagnose CPSS if pain persists for 6–12 months following spinal surgery.^33^ Yet, CPSS rats displayed the most significant pain hypersensitivity in the initial 2–3 weeks after rod removal and laminectomy. Although a rat’s 30-day life is equivalent to one human year,^31,32^ the relatively quick onset and limited duration of evoked pain hypersensitivity in CPSS rats suggest this model may partially mirror the progression of CPSS in humans. In addition, CPSS in patients involves multifactorial considerations, including emotional and social factors,^34^ posing challenges in fully replicating and analyzing these complexities in animal models.

Rats possess remarkable self-healing abilities.^35,36^ Their peripheral nervous system also shows greater resistance to injury, enabling faster recovery from nerve damage.^35^ Unlike humans, where nerve compression often exacerbates symptoms with activity, rats typically experience a quicker reduction in hypersensitivity following similar injuries, such as in DRG compression models.^36^ The differences in recovery between rats and humans are thought to stem from species-specific variations in healing processes, including enhanced angiogenesis, increased cell proliferation, and reduced inflammatory responses.^35,36^ Therefore, species-specific differences should be taken into consideration when assessing treatment outcomes in rats, especially when simulating clinical treatment protocols.

Among the various pain-related genes in the DRG, we assessed P2X7 and GFAP, which are mainly expressed in non-neuronal cells (e.g., satellite glial cells) in the ganglion, TRPA1, which is an important receptor extensively expressed in nociceptive DRG neurons and MOR which is a potential target on DRG neurons for inhibiting CPSS with peripherally acting MOR agonists. This represents an initial exploration into characterizing the molecular basis of this new CPSS model. There are many other receptors on DRG neurons that are important for pain. Understanding their transcriptional and translational roles in CPSS is essential for developing targeted therapies. Full-scale studies on the cellular and molecular mechanisms of CPSS are warranted, including comprehensive profiling of changes in gene and protein expression of other receptors in DRG neurons and glial cells, investigating the signaling pathways, and examining the effects of other analgesics used in clinical pain management (e.g., gabapentin, clonidine) to provide deeper insights into the pathophysiology of CPSS.

## Conclusion

The mechanisms underlying CPSS remain poorly understood, particularly those related to non-structural factors. We present a new model which mimics the natural progression of human CPSS from its onset to surgical intervention, while excluding potential structural factors. CPSS rats display a range of traits that mirror the intricate aspects of CPSS observed in humans, including the progression of the condition and its impact on function. This similarity facilitates deeper research into the underlying mechanisms and enables exploration of its treatment and prevention strategies. In addition, current findings highlight the potential of targeting MOR in DRG neurons and using peripherally acting MOR agonists for CPSS treatment.

## Methods

### Animals

Adult male and female Sprague-Dawley rats (10 weeks, 250–300 g, Harlan Bioproducts for Science, Indianapolis, IN) were housed under standard laboratory conditions with a 12-h light/dark cycle and free access to food and water. All experimental protocols received approval from the Johns Hopkins University Animal Care and Use Committee (Baltimore, MD, USA), adhering to the National Institutes of Health Guide for the Care and Use of Laboratory Animals to ensure ethical standards and minimize animal discomfort.

### Surgery

The development of CPSS model involves two steps. Step 1: Inducing LBP by compressing the DRG. Rats were anesthetized with a mixture of isoflurane (1.5%–2.0%, Abbott Laboratories, North Chicago, IL) and oxygen. The surgical area was shaved and sterilized with povidone-iodine. A 2.0 cm posterior midline incision was made at the lumbar region (L4-L6), using 11 sharp tip blades through the skin, fascia, and underlying muscle and the fascia. The left paravertebral muscles were stripped from the spinous process. The intervertebral foramen on the left side of the L5 vertebra was exposed, and a small blunt plastic rod (tip diameter: 1 mm; length: 6 mm) was slowly inserted with tweezers. We inserted the first 3–4 mm of the 6-mm rod into the foramina at an angle of 45° to the midline of the back, which would induce a chronic steady compression of the DRG and spinal nerve (Fig. 1a, Fig. S4). This effect mimics the intervertebral foramen stenosis and compression which may cause LBP in patients. Then, the surgical wounds and skin were sutured in anatomic order, with 4-0 PolysynTM (synthetic absorbable) sutures. Step 2: Patients with intervertebral foraminal stenosis and compression often require decompression spinal surgery, typically involving laminectomy. To mimic it, decompression surgery was conducted at 7 days after Step 1 surgery when rats had developed mechanical and heat hypersensitivities in the ipsilateral hindpaw (Fig. 1b–d). The procedure begins with a midline incision of 2–3 cm with the target vertebral body at the center, the paraspinal muscles were separated to expose the bony posterior elements. We first pulled out the plastic rod implanted in Step 1 to relieve the compression. Then, we performed a full laminectomy at the L5 spinal level to mimic the decompression surgery performed in patients. The laminectomy was performed with caution to keep the dura mater intact. The dura mater was then covered with the paraspinal muscles during suture. Finally, the surgical wounds and skin were sutured in anatomic order, with 4-0 PolysynTM sutures.

### Behavioral testing

#### Mechanical sensitivity test

Mechanical sensitivity was assessed by measuring the paw withdrawal threshold (PWT) to von Frey filament stimulation.^37^ Briefly, the von Frey filaments were applied to the mid-plantar area of each hind paw for 4 s to 6 s. If a positive response occurred (abrupt paw withdrawal, shaking, and licking), a smaller von Frey hair was applied next; if no response was observed, a higher force was applied. The test continued until (1) five stimuli were assessed after the first crossing of the withdrawal threshold or (2) the upper/lower limit of the von Frey hair set was reached before a positive/negative response was obtained. PWT was calculated using the formula from Dixon.^38^

#### Heat sensitivity test

Heat sensitivity was evaluated by measuring paw withdrawal latency (PWL) to radiant heat stimuli.^22^ Animals underwent 7 days of handling training prior to data collection and were subsequently acclimated to the testing environment for 30 min before each session. Animals were placed under a transparent plastic box on a glass floor, and radiant heat was applied to the hind paw using a plantar stimulator analgesia meter (IITC model 390, Woodland Hills, CA). After acclimatization, the latency for the animal to withdraw its hind paw in response to the heat was recorded. Radiant heat was applied to the mid-plantar area of each hind paw three times with 5-min intervals between trials. A cutoff time of 20 s was set to prevent tissue damage. Data from three trials were averaged for analysis.

#### Cold sensitivity test

The cold plantar assay assessed cold sensitivity.^39^ Animals were individually placed in clear acrylic containers with white opaque dividers on a glass plate and acclimated for 20 min before testing. Powdered dry ice in a cutoff 10-mL syringe was applied beneath the hind paw until withdrawal. The procedure was repeated every 5 min, alternating between paws, for a total of 4 trials, and the mean PWL was calculated. A cutoff time of 45 s was set to prevent tissue damage.

#### Open field test

The open field test evaluated locomotor activity and spontaneous exploration, following protocols described in previous studies.^8,39^ Rats were placed in a rectangular plastic chamber (73 × 45 cm) with a wall height of 33 cm for 10 min. Parameters including total distance traveled, number of center crossings, and distance traveled in the central area were analyzed using SMART 3 software (Panlab Harvard Apparatus, Barcelona, Spain) from video recordings.

#### Catwalk analysis

The Noldus CatWalk XT system assessed rat gait.^40^ After 4 weeks of acclimation and conditioning training, rats voluntarily traversed the platform while a camera positioned 42 cm below recorded foot placements. CatWalk software automatically classified each run, with independent researchers correcting misidentified steps. Stand time, max-intensity, and max-contact-area of hind paws were computed using CatWalk software. Data from three trials were averaged for analysis.

#### Rotarod test

The rotarod test assessed passive locomotor activity, following protocols described in a previous study.^41^ Rats were acclimatized and trained on a rotating rod (Ugo Basile, Italy) that accelerated from 0 to 30 r/min in 180 s. The duration (in seconds) that each animal remained on the accelerating rod without falling was recorded. Data from three trials were averaged for analysis.

### DRG neuronal culture and calcium imaging

The experiments were conducted as previously described.^42^ DRGs were harvested from rats and placed in cold DH10-20 medium (90% DMEM/F-12, 10% fetal bovine serum, penicillin [100 U/mL], and streptomycin [100 μg/mL]; Invitrogen, Waltham, MA). They were then treated with an enzyme solution (dispase [5 mg/mL] and collagenase type I [1 mg/mL] in Hanks’ balanced salt solution without Ca^2+^ or Mg^2+^) for 35 min at 37 °C. Following trituration, the supernatant-containing cells were filtered through a Falcon 40 µm (or 70 µm) cell strainer. Subsequently, the cells were centrifuged, resuspended in DH10 medium, and plated on poly-D-lysine-coated (0.5 mg/mL; Biomedical Technologies Inc, Madrid, Spain) and laminin-coated (10 μg/mL; Invitrogen) glass coverslips. They were then cultured in an incubator (95% O2 and 5% CO2) at 37 °C and utilized within 48 h. Neurons were loaded with Fura-2-acetomethoxyl ester (Molecular Probes, Eugene, OR) for 45 min in the dark at room temperature. After washing, cells were imaged at 340 and 380 nm excitation wavelengths to detect intracellular free calcium. Calcium imaging assays were conducted by an experimenter blinded to drug treatment.

### Whole-cell patch-clamp recording

Whole-cell patch-clamp technique was employed to investigate the electrophysiological and pharmacological characteristics of intrinsic membrane properties of DRG neurons obtained from adult rats.^9^ Following a 48–72 h incubation period, cultured DRG neuron preparations were transferred to a recording chamber (RC-22; Warner Instruments, Hamden, Connecticut, USA), mounted on an inverted microscope, and visualized using a bright-field imaging system (Eclipse TE2000-U; Nikon). Electrode tip impedance ranged from 2 to 4 MΩ and formed seal resistances >1 GΩ when filled with an internal recording solution. For patch-clamp recordings in small-diameter (<25 μm) DRG neurons, series resistance was maintained at <20 MΩ for ~1–2 min after achieving whole-cell configuration. Following a 1-min baseline recording to assess the stability of each evoked current, the drug was applied to the neurons using a six-channel perfusion valve control system (VC-6; Warner Instruments) for 2 min (duration of full bath exchange), followed by a 5-min washout with an external solution.

### Western blot

Tissues were lysed in radioimmunoprecipitation assay (RIPA) buffer containing protease and phosphatase inhibitor cocktail. The protein concentration of RIPA lysates was determined using a standard bicinchoninic acid protein assay. Samples (20 mg) were separated on a 4% to 12% Bis-Tris Plus gel and transferred onto a polyvinylidene difluoride membrane. Immunoreactivity was detected by enhanced chemiluminescence after overnight incubation with the indicated primary antibody (4 °C). Antibodies against TRPA1 (1:500; Novus Biologicals, NB110-40763SS), MOR (1:1 000; Neuromics, RA10104), P2X7 (1:1 000; Alomone, APR-004), and GFAP (1:50; ImmunoStar, AB572240) were chosen. Glyceraldehyde 3-phosphate dehydrogenase (GAPDH, 1:100 000; Millipore, Darmstadt, Germany) served as an internal control for protein loading. Image J was employed to quantify the intensity of immunoreactive bands on autoradiograms.

### Nissl staining and Immunocytochemistry (ICC) staining

On day 7 post-Step 2 surgery, contralateral and ipsilateral L5 DRGs were harvested from CPSS rats, paraffin-embedded, and sectioned at 8 μm thickness. Nissl staining was performed following the manufacturer’s protocol. Briefly, the sections were deparaffinized, stained for 15 min, dehydrated in absolute ethanol, cleared in xylene, and sealed with neutral gum. Microscopic inspection, image acquisition, and analysis were subsequently conducted. Immunocytochemistry was employed to detect caspase-3 and NeuN expression. Paraffin-embedded tissue sections were deparaffinized in xylene and rehydrated through an ethanol gradient (100%, 95%, 70%, and 50%, 5 min each) followed by rinsing in water. Antigen retrieval was performed, and endogenous peroxidase activity was reduced. The sections were then incubated overnight at 4 °C with the primary antibodies (Anti-NeuN, Servicebio, GB15138, 1:500 dilution; Anti-caspase-3, Servicebio, GB11532, 1:500 dilution). Following incubation, the sections were labeled with secondary antibodies and visualized. Images were captured using a Nikon light microscope (Tokyo, Japan).

### Drugs

Dermorphin [D-Arg2, Lys4] (1-4) amide (DALDA, US Biological Life Sciences, Salem, Massachusetts) was diluted in normal saline. For in vivo pain behavior tests, rats were administered intraperitoneal (i.p.) injections of DALDA at a dose of 5 mg/kg, based on previous findings.^8,9^ For in vitro experiments, a concentration of 5 μmol/L was used for whole-cell patch-clamp recording and calcium imaging tests.

### Statistical analysis

Statistical analyses were performed using GraphPad Prism 8.4.2 software. Parametric (two-tailed unpaired or paired *t*-tests) or non-parametric (Mann-Whitney test) methods were employed to compare the two groups. One-way ANOVA followed by the Bonferroni post hoc test was applied for comparisons involving three or more groups. Two-way mixed model ANOVA followed by Sidak’s multiple comparisons test was utilized for analyses involving different factors across multiple groups. Data are presented as mean ± SEM. A significance level of *P* < 0.05 was considered statistically significant in all tests.

## Supplementary information

Supplementary Figures

## Data Availability

Data sets generated from the current study will be available and shared with all communities of scientists upon request.
