# Supplementary material for: Characterizing a new rat model of chronic pain after spine surgery
Source: Bone Res. 2025 Mar 12;13:34. doi: 10.1038/s41413-025-00408-1 (PMC11904174; doi:10.1038/s41413-025-00408-1)
Supplement: Supplementary file 1 — Supplementary Figures [file 41413_2025_408_MOESM1_ESM.docx]

**Supplementary Figures.**


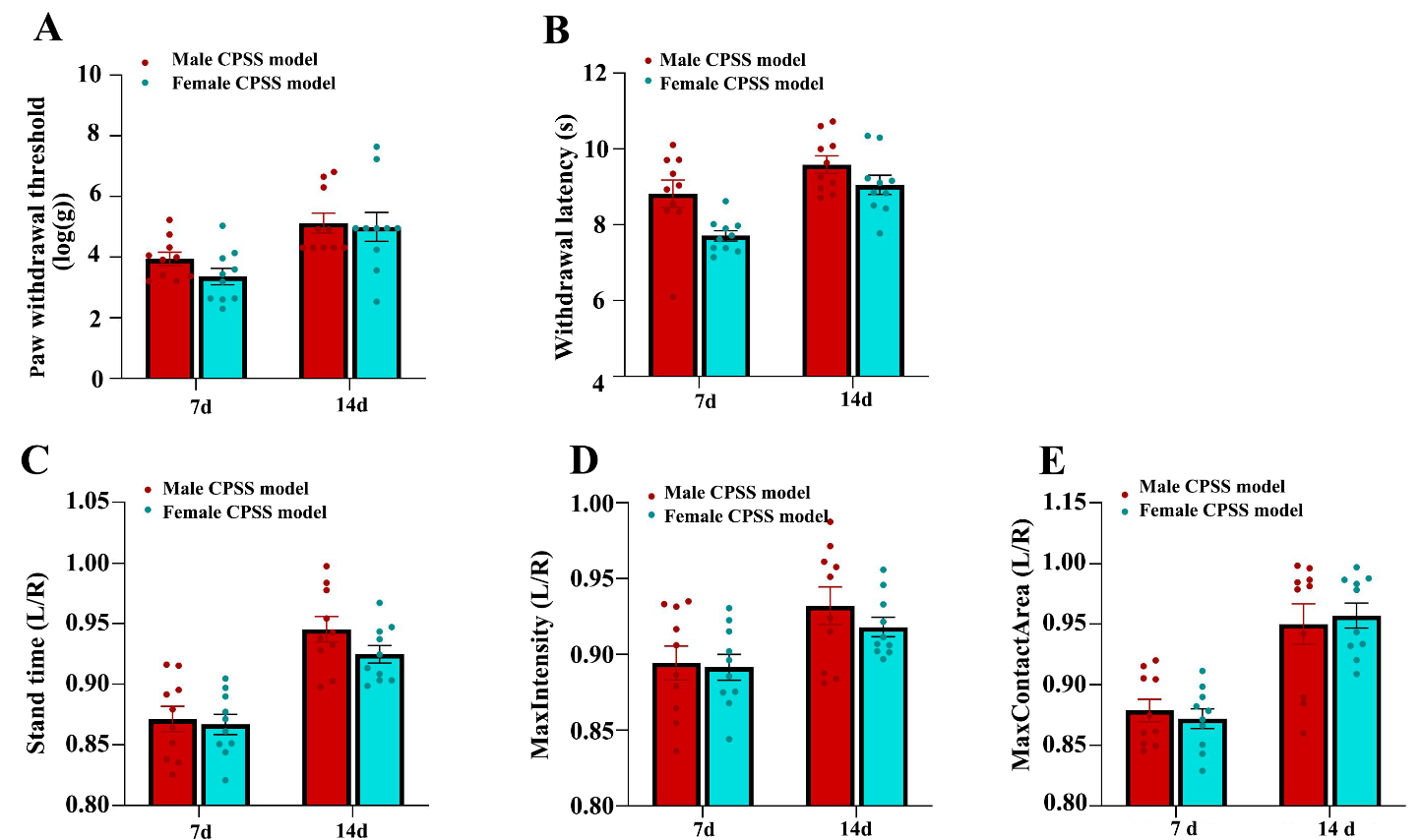


**Supplemental Figure 1. Comparing the behavioral changes between male and female CPSS rats. (A)** The ipsilateral paw withdrawal threshold (PWT) to mechanical stimuli in male and female CPSS rats at days 7 and 14 after surgery (n=10/group). **(B)** The ipsilateral paw withdrawal latency (PWL) to heat stimuli in male and female CPSS rats at days 7 and 14 after surgery (n=10/group). **(C-E)** The standing time (C), maximum intensity (D), and maximum contact area (E) in Catwalk analysis of male and female CPSS rats (n=10/group). One-way mixed model ANOVA followed by the Bonferroni post hoc test. Data are expressed as mean ± SEM.


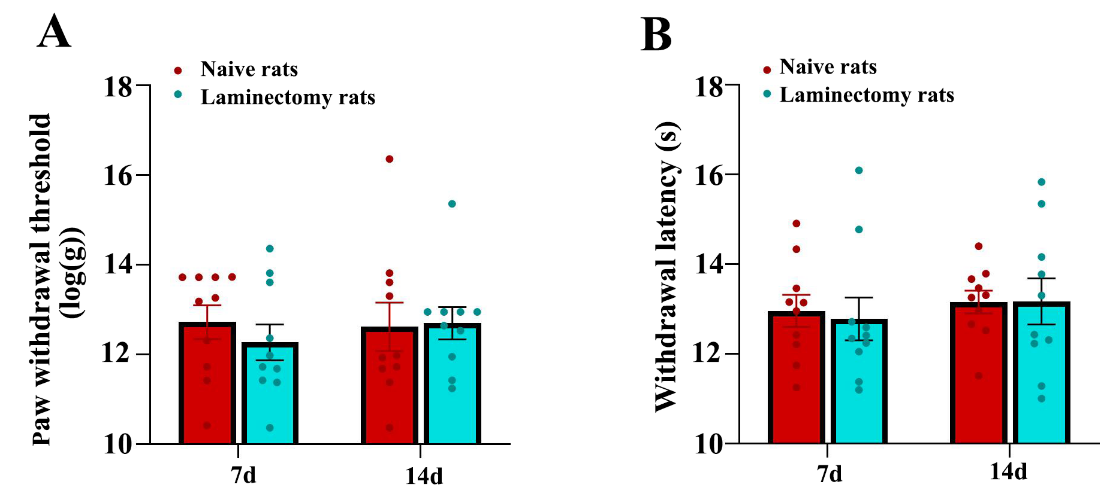


**Supplemental Figure 2. Effects of laminectomy alone on pain sensitivity in naïve rats. (A)** The paw withdrawal threshold (PWT) to mechanical stimuli in naive rats, and in rats at days 7 and 14 after L5 laminectomy surgery (n=10/group). **(B)** The paw withdrawal latency (PWL) to heat stimuli in naïve rats, and in rats at days 7 and 14 after laminectomy (n=10/group). One-way mixed model ANOVA followed by the Bonferroni post hoc test. Data are expressed as mean ± SEM.


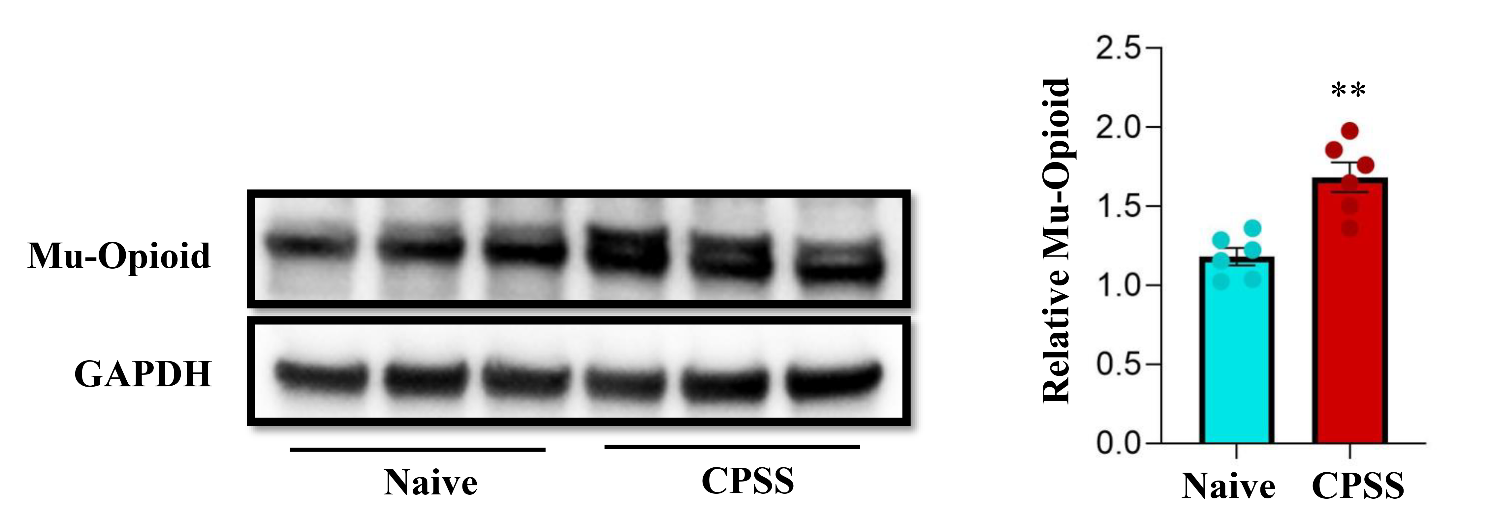


**Supplemental Figure 3. Increases in the expression of mu-opioid receptors in the ipsilateral L5 DRG of CPSS rats.** Left: Representative Western blot analysis images. Right: The quantification of the levels of mu-opioid receptor in the ipsilateral L5 DRGs of CPSS rats at day 7 after Step 2 surgery, compared to that of naive rats (n=6/group). Data are expressed as mean ± SEM. Unpaired t-test. ***P*<0.01.


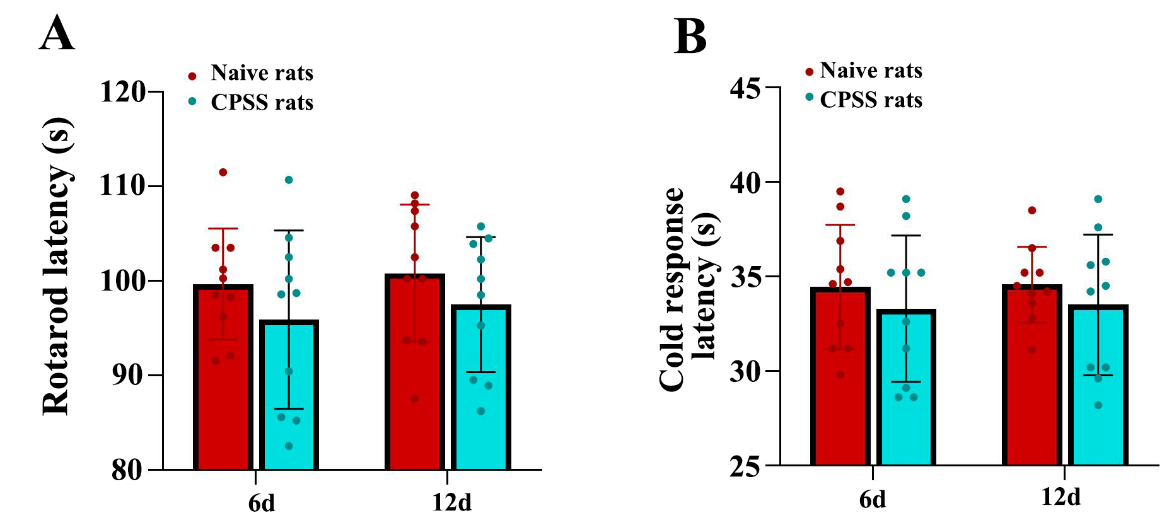


**Supplemental Figure 4. Rotarod test and cold sensitivity test in CPSS rats. (A)** In the rotarod test, there were no significant differences in fall latency between naive and CPSS rats on day 6 and day 12 after surgery (n=10/group). **(B)** Paw withdrawal latency (PWL) to cold stimuli in naive and CPSS rats at days 6 and 12 after surgery (n=10/group). One-way mixed model ANOVA followed by the Bonferroni post hoc test. Data are expressed as mean ± SEM.


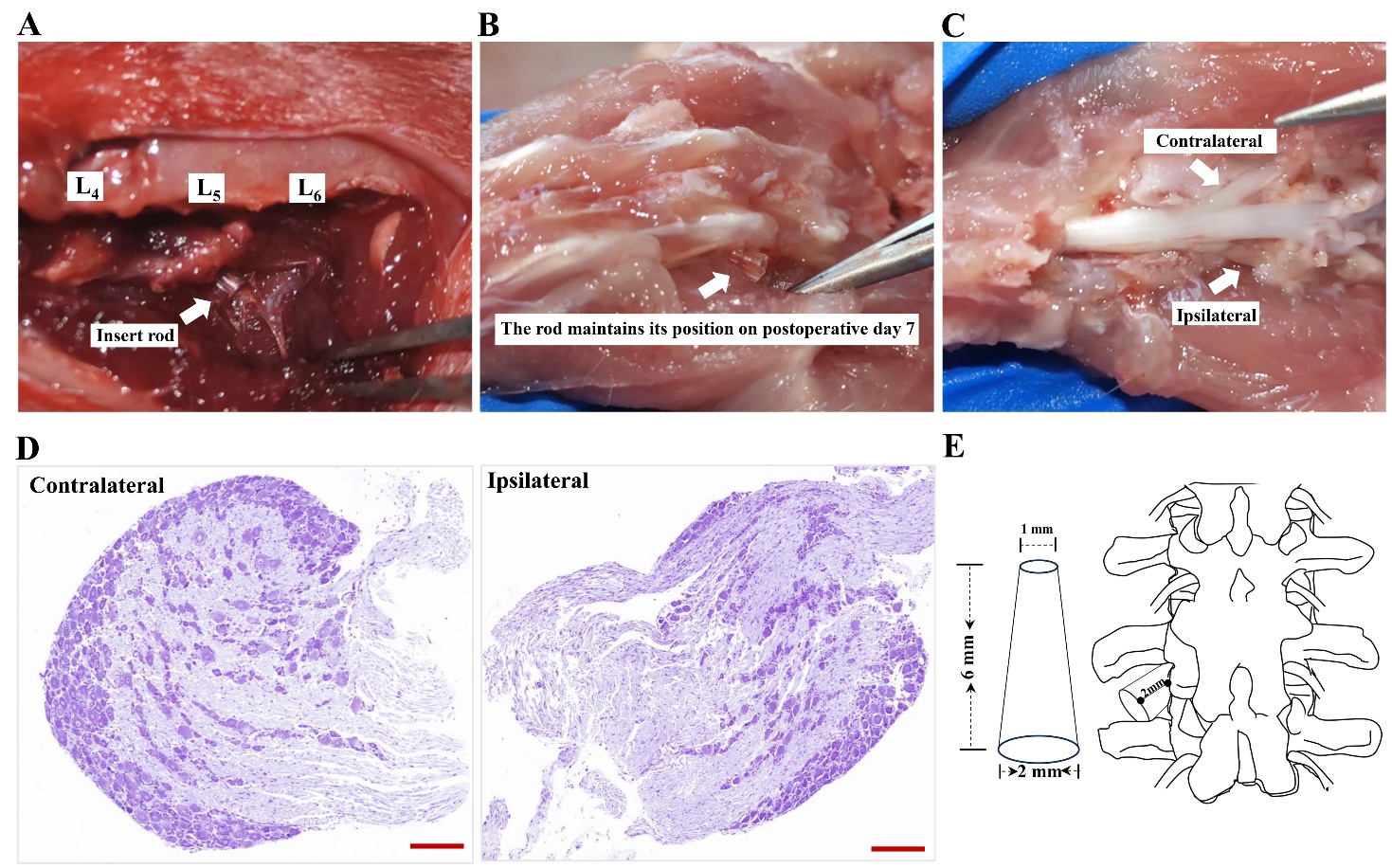


**Supplemental Figure 5.** **Example images of the inserted rod and Nissl staining of the L5 DRGs. (A)** An intraoperative image shows the rod inserted into the L5 intervertebral foramen of the rat. **(B)** Postoperative day 7 image demonstrating that the rod remained in its original position. **(C)** After a full laminectomy to expose the neural tissue on both sides, an image showing a clear sign of compression in the ipsilateral L5 DRG. **(D)** Representative Nissl staining images of the contralateral and ipsilateral L5 DRG in the same rat on day 7 after rod insertion. Scale bar: 100 µm. **(E)** Left: The diagram illustrates the parameters of the rod. Right: a simulation diagram of the rod after being inserted into the intervertebral foramen.


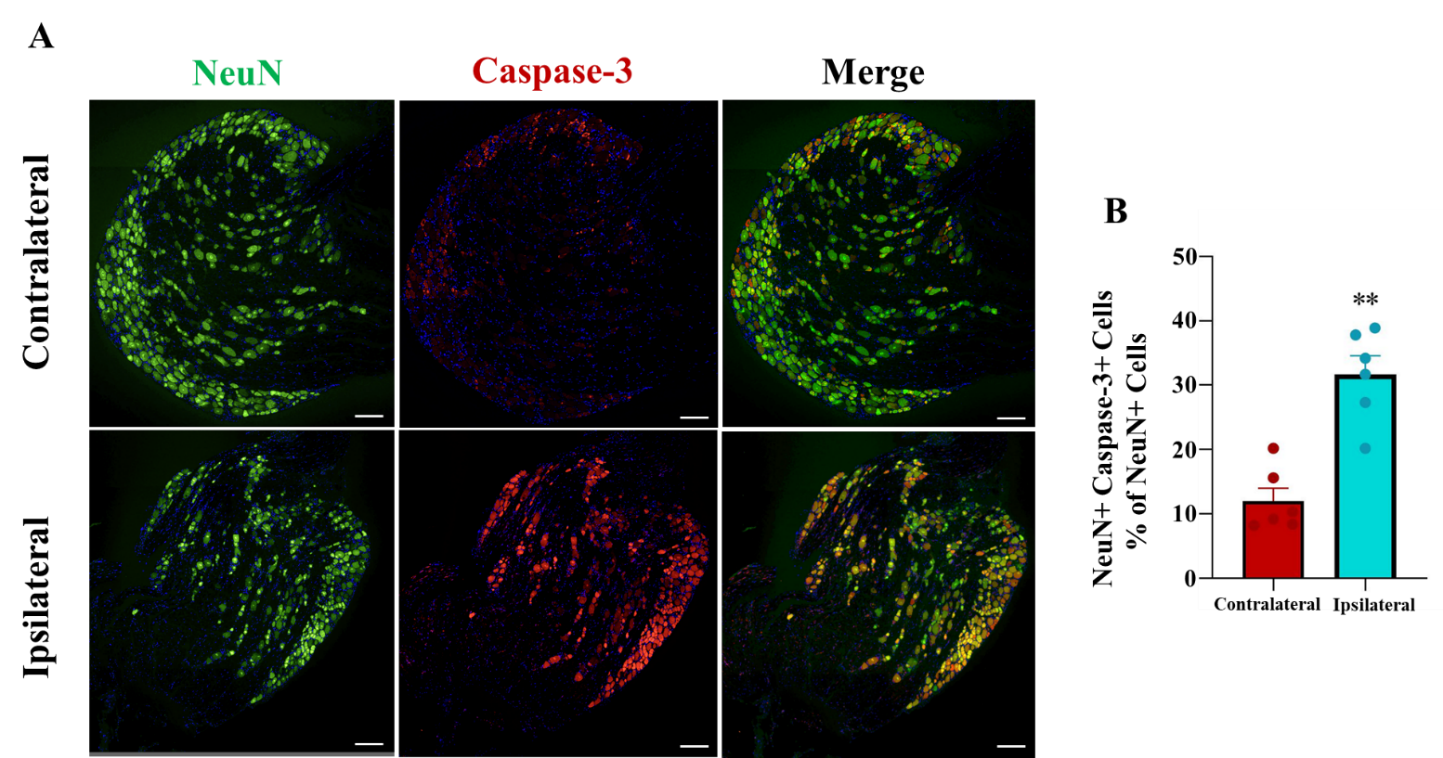


**Supplemental Figure 6. Increased marker of neuronal injury in the ipsilateral L5 DRG of CPSS rats. (A)** Representative images of double-labeling of caspase-3 and NeuN in the contralateral and ipsilateral L5 DRG removed from a CPSS rat on day 7 after Step 2 surgery. Scale bar: 100 µm. **(B)** Quantification of the percentage of NeuN^+^/caspase-3^+^ double-labeled cells in total NeuN^+^ cells on each side (n=6/group). Data are presented as mean ± SEM, Unpaired t-test. ***P*<0.01.
